# A new childhood ALL case with an extremely complex karyotype and acute spontaneous tumor lysis syndrome

**DOI:** 10.1186/s13039-020-00512-3

**Published:** 2020-09-11

**Authors:** Abdulsamad Wafa, Rami A. Jarjour, Doaa Alolabi, Thomas Liehr, Othman Hamdan, Joana B. Melo, Isabel M. Carreira, Moneeb A. K. Othman, Walid Al-Achkar

**Affiliations:** 1 Department of Molecular Biology and Biotechnology, Human Genetics Division, Atomic Energy Commission, Damascus, Syria; 2 Department of Hematology, Damascus Children University Hospital, Ministry of High Education, Damascus, Syria; 3 Jena University Hospital, Institute of Human Genetics, Jena, Germany; 4 Cytogenetics and Genomics Laboratory, Faculty of Medicine, University of Coimbra, Coimbra, Portugal; 5 CIMAGO-Center of Investigation on Environment Genetics and Oncobiology, Faculty of Medicine, University of Coimbra, Coimbra, Portugal

**Keywords:** Acute lymphoblastic leukemia (ALL), Complex karyotype (CK), Molecular cytogenetics, Array comparative genomic hybridization (aCGH), Tumor lysis syndrome (TLS), Prognostic factors

## Abstract

**Background:**

B cell precursor acute lymphoblastic leukemia (B-ALL) is the most common malignancy of childhood, with, after corresponding treatment, an overall complete remission rate of 90%. Approximately 75% of B-ALL cases harbor recurrent abnormalities, including so-called complex karyotypes (CK). Tumor lysis syndrome (TLS) is a metabolic abnormality which may arise during cancer therapy and also, extremely rarely, as spontaneous TLS before initiation of chemotherapy in patients with ALL.

**Case presentation:**

Here we report a 9-year-old male, diagnosed with a de novo pre-B-ALL according to the WHO classification. Cytogenetic, molecular cytogenetic approaches and array comparative genomic hybridization analyses revealed a unique CK involving five chromosomes. It included four yet unreported chromosomal aberrations: a der(11)t(7;11)(p22.1;q24.2), a der(18)t(7;18)(q21.3;p11.22), del(11)(q24.2q25) and dup(18)(q11.1q23). Unfortunately, the patient died 3 months after the initial diagnosis.

**Conclusions:**

To the best of our knowledge, a comparable childhood ALL case was not previously reported. Thus, the combination of the here seen chromosomal aberrations in childhood primary ALL seems to indicate for an extremely adverse prognosis.

**Electronic supplementary material:**

The online version of this article (10.1186/s13039-020-00512-3) contains supplementary material, which is available to authorized users.

## Background

B-cell precursor acute lymphoblastic leukemia (B-ALL) is the most common malignancy of childhood, representing ~ 80% of ALL cases [1, 2]. B-ALL patients have a favorable prognosis with an overall complete remission rate of 90% for children and adolescents between 1 and 15 years of age [3]. About 60% of B-ALLs harbor recurrent chromosomal abnormalities (including numerical and structural changes), which are detectable by banding cytogenetics. Those recurrent alterations can be used to define ALL specific subgroups, risk stratifying markers, predictors of clinical prognosis, and are essential for therapeutic planning, implementation of targeted therapy and sensitive monitoring of treatment response [4, 5]. In addition, targeted therapies have been developed for patients with *BCR/ABL* genes rearrangement; also immunochemotherapy has proven to be effective in those patients with a translocation t(8;14), t(8;22), or t(2;8) [6].

A complex karyotype (CK) has been generally classified as ≥ 3 unrelated (acquired) chromosomal abnormalities in a patients’ genome; CKs are predictive of poor outcomes in ALL [7, 8]. Interestingly CK in B-ALL is defined with ≥ 5 abnormalities [8]. CKs have been incorporated into the definition of high-risk ALL and many studies have suggested new definitions based on affected regions or types of aberrations [9, 10]. However, their prognostic significance has not been consistently validated in large series.

Tumor lysis syndrome (TLS) is a complication that can occur during the treatment of cancer. It is characterized by a metabolic abnormality including hyperuricemia, hyperkalemia, hyperphosphatemia and hypocalcemia. The latter may occur due to rapid lysis of tumor cells and it leads to severe renal impairment, cardiac arrhythmia and/or seizure and death [11, 12]. It is one of the oncologic emergency situations in patients with (potentially) lethal hematological and other malignancies [13]. Cytolysis of cancerous cells can be caused by chemotherapy or it can occur spontaneously [14–16]. Spontaneous TLS is a rare occurrence, and it may result in more severe clinical outcomes due to lack of benefit from pre-treatment [17].

We present here for the first time a yet unreported CK event involving five chromosomes in a childhood ALL case presenting with acute spontaneous TLS at diagnosis.

## Case presentation

A 9-year-old male patient with congenital adrenal hypertrophy at birth and treated with hydrocortisone and floudorocortisone suffered from abdominal and dorsal pain with vertigo and sweating without fever. Abdominal ultrasonography showed swollen appendix. Moreover, he had severe anemia (hemoglobin value was 2.7 g/dl). Therefore, he received blood transfusion and the parents noticed subsequently a mass in the neck. Appendectomy was performed but the symptoms were not resolved. The severity of pain increased particularly in chest, neck and head.

The patient was admitted to the Children’s Hospital in Damascus (day 1—Additional file 1: Table 1). Ultrasonography showed that he had urinary retention. Free fluid in Morison’s and splenorenal pouch and medium free fluid in the pelvic and bilateral pleural transfusion were diagnosed, too. He had severe abdominal pain, chills, pallor without fever, a bruise on the right thigh (5 × 3 cm), several lymphadenopathies (submandibular, bilateral sternocleidomastoidal (2 × 3 cm), supraclavicular and bilateral subaxillary (right 1 × 1 cm and left 0.5 × 0.5 cm), no splenomegaly, normal renal size and a heart rate of 100 beats per min. The patient presented TLS symptoms at the same time when being admitted to the Children’s Hospital. Bone marrow (BM) aspiration revealed 59% of blasts. Flow cytometric (FCM) analysis classified this case as pre-B-ALL. The patient was given treatment for BFM-NHL block AA (1989) induction chemotherapy protocol just D1, later the chemotherapy was stopped because the patient developed neutropenia and acute renal failure (for more details see Table 1).

**Table 1:** Results of aCGH. Summary of CNAs detected by aCGH

Chr.	Start–end band	Genomic position: start–end GRCh37/hg19	Variant type	Size (Mb)	COSMIC census cancer gene(s) within the region
7	p22.3p22.1	45,130–4,642,192	1 copy gain	4.6	*CARD11*
7	q21.3q36.3	96,556,335–159,128,556	1 copy gain	62.6	*TRRAP, CUX1, CREB3L2, MET, POT1, SND1, SMO, TRB, TRIM24, KIAA1549, BRAF, FAM131B, CNTNAP2, EZH2, KMT2C, MNX1*
11	q24.2q25	125,246,792–134,945,165	1 copy loss	9.7	*FLI1, KCNJ5*
18	p11.22p11.21	9,095,620–14,089,409	1 copy gain	4.99	
18	q11.1q23	18.550.472–78.012.829	1 copy gain	59.5	*ZNF521,SS18, SETBP1, SMAD2, SMAD4, DCC, MALT1, BCL2, KDSR,*

Approximately 2 months after initial diagnosis he died in the Children’s Hospital in Damascus due to respiratory and cardiac arrest, neutropenia, septicemia and renal failure; no autopsy was performed. His mother agreed with scientific evaluation of the case and the study was approved by the ethical committee of the Atomic Energy Commission, Damascus, Syria.

Banding cytogenetics analysis on unstimulated BM sample was performed according to standard procedures [18] prior of chemotherapy. Karyotype was classified according to the International System for Human Cytogenomic Nomenclature [19].

**Fig. 1:**
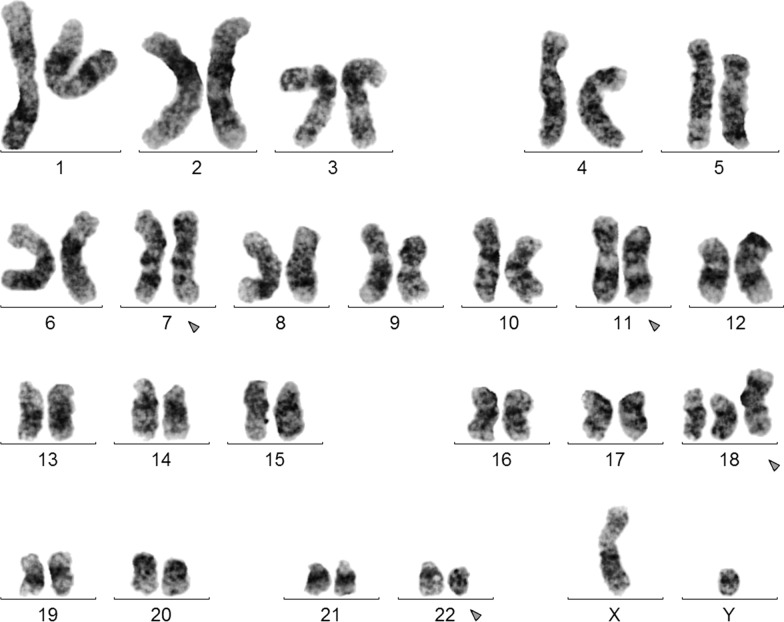
GTG-banding revealed a complex karyotype multiple numerical and or structural rearrangements

Prior to chemotherapy treatment: GTG-banding cytogenetics revealed the following karyotype: 48,XY,t(8;22)(?;?),+mar, +19 [17]/47,XY,t(8;22)(?;?),+mar [3] (Fig. 1).

Molecular cytogenetics approaches revealed the karyotype 48,XY,der(8)t(8;22) (q24;q11),der(11)t(7;11)(p22.1;q24.2),+der(18)t(7;18)(q21.3;p11.22),+19[17]/47,XY,der(8)t(8;22) (q24;q11),der(11)t(7;11)(p22.1;q24.2),+der(18)t(7;18) (q21.3;p11.22) [3]. Further analyses failed on the available material. Thus, genomic DNA was extracted from BM cells prior to chemotherapy treatment and aCGH was performed using the Agilent Sure Print G3 Human Genome Microarray 180 K as previously described [20]. The aCGH analysis revealed different genomic imbalances (Table 1): a loss of 9.7 Mb in the region of 11q24.2q25 between the positions 125,246,792 and 134,945,165 (GRCh37/hg19) including two COSMIC census cancer genes (https://cancer.sanger.ac.uk/census). Besides, gains of copy numbers were detectable in 7p22.3p22.1 at positions 45,130 and 4,642,192 including one COSMIC census cancer gene, 7q21.3q36.3 at positions 96,556,335 and 159,128,556 including 16 COSMIC census cancer genes, 18p11.22p11.21 at positions 9,095,620 and 14,089,409 (no COSMIC census cancer gene identified), and 18q11.1q23 at positions 18,550,472 and 78,012,829 including 9 COSMIC census cancer genes (Table 1).

Immunophenotyping was performed on the BM specimen prior and after chemotherapy treatment using a general panel of fluorescent antibodies against antigens typical for different cell lineages and cell types [21]: CD1a, CD2, CD3, CD4, CD5, CD8, CD10, CD11b, CD11c, CD13, CD14, CD15, CD16, CD19, CD20, CD22, CD23, CD32, CD33, CD34, CD36, CD38, CD41a, CD45, CD56, CD57, CD64, CD79a, CD103, CD117, CD123, CD138, CD209, CD235a and CD243; In addition to antibodies to Kappa and Lambda light Chains, IgD, sIgM, and HLA DR. All antibodies were from BD Biosciences. Flow cytometric data acquisition and analysis were conducted [22]. FCM analysis of BM specimen prior to chemotherapy treatment characterized this case as pro-B ALL according to the WHO classifications. The abnormal cell population (75% of tested cells) was positive for CD45^dim^, CD19, TdT, CD10, CD20 and HLA DR. Blast cell population was negative for CD34, CD79a, T cell linage and myeloid linage markers.

## Discussion and conclusions

To the best of our knowledge, we report here the first case of a childhood patient with a pre-B-ALL presenting with a yet unreported CK involving five chromosomes, with acute spontaneous TLS at diagnosis.

According to Mitelman Database of Chromosome Aberrations in Cancer [23], there are 1899 ALL cases documented. Among those, 17 cases of ALL with translocation t(8;22)(q24;q11), 29 cases with translocation t(7;11) involving short and/or long arms of both chromosomes, one case of ALL with dup(7)(q21q36), and 2 cases of ALLs with dup(18)(p11). In addition, the chromosomal bands 8q24, 22q11, 7p22, 7q21, 7q36,11q24, 11q25, 18p11, 18q11 and 18q23 are involved in chromosomal rearrangements in 198, 1,284, 80, 32, 68, 40, 50 56, 32, and 29 cases, respectively [23]. Interestingly, translocation der(11)t(7;11)(p22.1;q24.2),der(18)t(7;18) (q21.3;p11.22), del(11)(q24.2q25) and dup(18)(q11.1q23) have never been described in ALL cases. To the best of our knowledge, a combination of all these rearrangements in one ALL case at diagnosis was not previously reported yet, also [23].

CK was defined as a karyotype showing 5 or more unrelated chromosomal abnormalities in ALL cases with the absence of established translocations (t[9;22], t[v;11q23], t[1;19], t[8;14], and t[14q32])[8]. Moorman et al. [8] demonstrated those ALL patients with CK ≥ 4 or more unrelated chromosomal abnormalities had a poor outcome in terms of overall survival and event-free survival, with most of the relapses occurring in the first 2 years after diagnosis. However, Motll’o et al. [6] showed that CK was not associated with an adverse prognosis in adult ALL patients treated with risk-adapted or subtype-oriented protocols.

TLS is a metabolic derangement characterized by hyperuricemia, hyperkalemia, hyperphosphatemia, hypocalcemia and acute kidney injury. It is a medical emergency and typically occurs after or before chemotherapy [11–13]. Risk factors for the development of TLS are large tumor burden, extensive metastasis, renal infiltration and high rate of cell turnover etc. [24]. TLS occurs more frequently in hematological malignancies than in solid tumors. The highest risk of developing TLS is observed in patients with lympho-proliferative disorders especially with high proliferative rate and high tumor sensitivity to chemotherapy, like B-cell ALL and Burkitt’s lymphoma [25, 26]. Tumor burden reflected by serum lactate dehydrogenase (LDH) level, initial WBC count, tumor size, and extensive bone marrow involvement are the main predictor for development of TLS in these patients [12]. However, the lymphoblastic lymphoma patients with LDH ≥ 2 × ULN classified as high-risk group [26]. Our patient had elevator LDH value 2,950 U/l at diagnosis.

Moreover, TLS is one of the most significant causes of acute renal failure (ARF) in cancer patients. ARF that results from acute TLS is usually oligoanuric and is multifactorial in etiology [27]. The most important cause of ARF is precipitation of uric acid in the tubulus of kidney due to hyperuricemia caused by increased turnover of nucleic acids [28]. Hyperphoshatemia contributes to renal failure by deposition of calcium phosphate complex in the renal interstitium [27, 28]. Our patient presented with non-oliguric ARF with extreme hyperuricemia and hyperphosphatemia. Diagnosis of spontaneous acute TLS that results from ALL was made as there was no contributing factor for development of renal failure.

ALL patients presenting with acute spontaneous TLS is very rare. Since acute TLS is a medical emergency with a high mortality rate, early recognition and prevention especially in the patients at high risk for this syndrome is essential. Patients with acute TLS must be treated with aggressive intravenous hydration with saline fluids and loop diuretics to maintain adequate urine output [27, 28]. Our patient was in non-oliguric ARF and was treated initially with aggressive intravenous hydration and allopurinol.

According to the literature, acute spontaneous TLS before initiation of chemotherapy in patients with ALL is extremely rare. The exact mechanisms for development of this syndrome in our patient are uncertain. We report here the first case of a childhood patient with pro-B-ALL presenting with a yet unreported complex karyotype involving five chromosomes with acute spontaneous TLS at diagnosis. Adverse outcome of the case may be partially caused by complicated of ARF and chromosomal aberrations.

## Supplementary information

**Additional file 1: Table 1.** Clinical history of the patient together with diagnostic results and treatment.

## Data Availability

All relevant data and material is included in this publication.

## Abbreviations

aCGH: Array comparative genomic hybridization
aMCB: Array-proven multicolor banding
ALL: Acute lymphoblastuc leukemia
AML: Acute myeloid leukemia
ARF: Acute renal failure
BM: Bone marrow
CK: Complex karyotype
DAPI: 4′,6-Diamino-2-phenylindole
M-FISH: Multiplex-fluorescence in situ hybridization
HGB: Hemoglobin level
LDH: Lactate dehydrogenase
PB: Peripheral blood
PLT: Platelet count
RBC: Red blood cells
TLS: Tumor lysis syndrome
WBC: White blood cells
WCP: Whole chromosome painting
WHO: World Health Organization
