# Supplementary material for: A new childhood ALL case with an extremely complex karyotype and acute spontaneous tumor lysis syndrome
Source: Mol Cytogenet. 2020 Sep 11;13:44. doi: 10.1186/s13039-020-00512-3 (PMC7488544; doi:10.1186/s13039-020-00512-3)
Supplement: Supplementary file 1 — Additional file 1: Table 1. Clinical history of the patient together with diagnostic results and treatment. [file 13039_2020_512_MOESM1_ESM.docx]

**Suppl. Table 1**

| **Day** | **Symptoms** | **Lab. test results** | **Management** |
| --- | --- | --- | --- |
| 1  (admission at hospital) |  | Peripheral blood (PB) showed: white blood cell (WBC) 11.26 x10^9^/l (neutrophils 40%, lymphocytes 25%)  anemia (Hgb = 6.9 g/dl),  and thrombocytopenia (Plt = 17 × 10^9^/l)  Serum biochemistry analyses:  Calcium (Ca^+2^) 9.9 mmol/l (normal value 8.5-10.3 )  LDH 2950 U/l (normal level <460)  Phosphor 4.4 mg/dl (2.7-6)  uric acid (UA) 16.3 mg/dl (normal value 3.5-7)  Cr 1.069  Creatinine (creat.)1.07 umol/l (normal 45-120)  Urea 54 mmol/l (normal 10-50)  Sodium (Na^+^) 140 mmol/l (normal135-148)  Potasium (K^+^) 3.5 mmol/l (3.5-5.2)  Urine analysis: UA +++, HgB ++++, RBC 20-25 cells, WBC 3-4 cells | Blood transfusion and antibiotics:  WBC 5.1 ( neutrophils 70%) x10^9^/l  HgB 12.7 g/dl  Plt 174× 10^9^/l  Ca^+2^ 0.8 mmol/l  P 5.9 mg/dl  UA 9.2 mg/dl  Creat. 2.8 umol/l  Urea 57 mmol/l  Na^+^ 134 mmol/l  K^+^ 3 mmol/l |
| 11 days later | Hepatosplenomegaly Right renal enlargement  Pelvic free fluid | BM aspirate showed: 59% of lymphoblast | Blood transfusion and antibiotics  Allopurinol (Zyluric)  Morphin sulphate  WBC 10.7 x10^9^/l (neutrophils 37%)  HgB 6.7 g/dl  Plt 7× 10^9^/l  LDH 2353 U/l  P 4.2 mg/dl  UA 16.8 mg/dl |
| 12 days later | Hepatosplenomegaly Right renal enlargement  Cervical Lymph node enlargement (right 3 cm) (left 1.1 cm)  Bilateral pleural effusion.  Ascites  Fever (39.5-40 C˚)  Acute Renal failure  Heart rate 90/min | FCM of BM: Pre-B-ALL  Karyotyping: complex karyotype   Renal clearance ratio: 29% | Amlodipine (Norvask)  Carbapenems (Meropenem)  Alkalization 70Eq/l  Hydration 2 l/m^2^  Blood transfusion |
| 18 days later | Heart rate 130/min  Fever 38.5-39  Brain CT scan: normal  bruise on the right elbow and pubic region  Gingival bleeding  Edemas in the lower extremities and hands  Jugular Venous Pressure  Ultrasonography: right and left renal enlargement (grade I) (3cm)  Free fluid in Morison's pouch and medium free fluid in the pelvic  Two Lymph node enlargement above Morison's pouch (1.6x5.8 cm) | WBC 9.4 x10^9^/l (neutrophils 37%)  HgB 7 g/dl  Plt 19× 10^9^/l  Ca^+2^ 7 mmol/l  P 7.9 mg/dl  UA 3.2 mg/dl  Creat. 1.6 umol/l  Urea 52 mmol/l  Na^+^ 143 mmol/l  K^+^ 2.2 mmol/l  Urinalysis: Urate ++, HB +++, WBC 2-3  CSF aspiration: no cells, protein 2.9  PT 65%  PTT 29 sec  True GFR 33.9 ml/l | Lazix 2mg/kg  Predlone 0.5 mg/kg  MTX 12mg  Amlodipine (Norvask)  Carbapenems (Meropenem)  Alkalization 70Eq/l  Hydration 2L/m2  Blood transfusion  WBC 5 x10^9^/l (neutrophils 21%)  HgB 7 g/dl  Plt 23× 10^9^/l  Ca^+2^ 7.25 mmol/l  P 17 mg/dl  UA 8.2 mg/dl  Creat. 2 umol/l  Urea 153 mmol/l  Na^+^ 139 mmol/l  K^+^ 2.8 mmol/l |
| 30 days later | Melina  Heart rate 140/min  Edemas in the lower extremities and hands  Bilateral pleural effusion  Aorta dilatation 26x42 mm  FS 38%  EF 69%  Neutropenia  Fever 38-39.5  Crackles in the right And left lungs  Vomiting  Heart rate 100/min | WBC 0.9 x10^9^/l (neutrophils 27%)  HgB 6.8 g/dl  Plt 24× 10^9^/L  Ca^+2^ 7.4 mmol/l  P 10.5 mg/dl  UA 17.4 mg/dl  Creat. 1.6 umol/l  Urea 224 mmol/l  Na^+^ 159 mmol/l  K^+^ 2.3 mmol/l  Blood osmotic pressure 357  Urine osmotic pressure 2  Partial excretion of Na^+^ 7.2% (0.3-1.6%)  Partial excretion of K^+^ 33% (10-30%)  Arterial gases: PH 7.08, Pco2 32, PO2 158, Hco3 30.6, Sat 99% | Treatment: As above (31/1/2019) + Tenam  Blood transfusion:  WBC 0.6 x10^9^/l (neutrophils 70%)  HgB 7.9 g/dl  Plt 16× 10^9^/l  Ca^+2^ 1.67 mmol/l  P 5.4 mg/dL  UA 6.1 mg/dl  Creat. 3.5 umol/l  Urea 167 mmol/l  Na^+^ 166 mmol/l  K^+^ 2.1 mmol/l  Renal Dialysis twice a week  Venctrestine+ predlone 2mg/kg+lasix 2 mg/kg  WBC 1.2 x10^9^/l (neutrophils 15%)  HgB 8.8 g/dl  Plt 42× 10^9^/l  Ca^+2^ 0.5 mmol/l  P 7.3 mg/dl  UA 3.2 mg/dl  Creat. 2.8 umol/l  Urea 199 mmol/l  Na^+^ 150 mmol/l  K^+^ 2.5 mmol/l |
| 38 days later | Neutropenia  Epigastric pain  Cellulitis in both arms  Fever 39.5 C˚  Bilateral pleural effusion  Heart rate 100/min | WBC 5.1 x10^9^/l (neutrophils 48.2%)  HgB 9.3 g/dl  Plt 336 × 10^9^/l  Ca^+2^ 1.12 mmol/l  P 7.8 mg/dl  UA 3.2 mg/dl  Creat. 1.01 umol/l  Urea 70.2 mmol/l  Na^+^ 150 mmol/l  K^+^ 2.5 mmol/l  CSF aspiration: no cells, protein 0.12 | D1 of BFM-NHL block AA (1989)  Neupogen 5mg/kg  Ranitidine  Septrine  Linozolide  Blood transfusion:  WBC 1 x10^9^/l (neutrophils 33%)  HgB 10.8 g/dl  Plt 110 × 10^9^/l  Na^+^ 129 mmol/l  K^+^ 3.2 mmol/l  Ca^+2^ 0.14 mmol/l  Urea 88.8 mmol/l  Creat. 1.44 umol/l  P 5.2 mg/dl  UA 5 mg/dl  Total protein (TP) 7.9 g/dl (normal 6.6-8.7 )  Albumin (Alb) 4 g/dl (normal 3.8-5.4)  ALT 23.2 U/l (normal 10-40)  AST 30 U/l (normal 10-40)  GGT 67 U/l ( normal 5-49)  Amylase 99 U/l ( normal (30-90)  PT 55%  PTT >100 sec |
| 52 days later | Dilated gallbladder 4.5x8.9 mm  left renal enlargement (grade I)  dilated bowel loops by ultrasound 2.8 cm  Abdominal pain and green vomit  Recurrent diarrhea  Fever 39-40 C˚  Aphthus  Gingival bleeding  Heart rate 95/min  Ultrasonography: masses in segment VI of the liver 3x2.7 cm and two masses 3.6x3.7 cm on the anterior hepatic margin  Crackles | WBC 0.5 x10^9^/l (neutrophils 22.6%)  HgB 7.4 g/dl  Plt 27 × 10^9^/l  Na^+^ 130 mmol/l  K^+^ 3.4 mmol/l  Ca^+2^ 1.1 mmol/l  Urea 66 mmol/l  Creat. 1.19 umol/l  P 7.1 mg/dl  UA 2.1 mg/dl | D2 was not applied because of the neutropenia  Neupogen 5mg/kg  Septrine  Linozolide  Flounoconazole  Blood transfusion  WBC 0.8 x10^9^/l (neutrophils 33%)  HgB 5.6 g/dl  Plt 15 × 10^9^/l  Na^+^ 151.5 mmol/l  K^+^ 3.5 mmol/l  Ca^+2^ 1.1 mmol/l  Urea 75 mmol/l  Creat. 0.9 umol/l  TP 4.4 g/dl  Alb 2.1 g/dl |
| 54 days later | Brain MRI: dilatation of ventricles and sulci | Approximately 2 months after initial diagnosis he died due to respiratory arrest and heart, neutropenia, septicemia and renal failure. | |
